# Estimating Typhoid Fever Risk Associated with Lack of Access to Safe Water: A Systematic Literature Review

**DOI:** 10.1155/2018/9589208

**Published:** 2018-07-04

**Authors:** Vijayalaxmi V. Mogasale, Enusa Ramani, Vittal Mogasale, Ju Yeon Park, Thomas F. Wierzba

**Affiliations:** ^1^Epidemiology Unit, International Vaccine Institute, Seoul, Republic of Korea; ^2^Policy and Economic Research Department, International Vaccine Institute, Seoul, Republic of Korea; ^3^Biostatistics and Data Management Department, International Vaccine Institute, Seoul, Republic of Korea; ^4^Development and Delivery Unit, International Vaccine Institute, Seoul, Republic of Korea; ^5^PATH, 455 Massachusetts Avenue NW, Suite 1000, Washington, DC, USA

## Abstract

**Background:**

Unsafe water is a well-known risk for typhoid fever, but a pooled estimate of the population-level risk of typhoid fever resulting from exposure to unsafe water has not been quantified. An accurate estimation of the risk from unsafe water will be useful in demarcating high-risk populations, modeling typhoid disease burden, and targeting prevention and control activities.

**Methods:**

We conducted a systematic literature review and meta-analysis of observational studies that measured the risk of typhoid fever associated with drinking unimproved water as per WHO-UNICEF's definition or drinking microbiologically unsafe water. The mean value for the pooled odds ratio from case-control studies was calculated using a random effects model. In addition to unimproved water and unsafe water, we also listed categories of other risk factors from the selected studies.

**Results:**

The search of published studies from January 1, 1990, to December 31, 2013 in PubMed, Embase, and World Health Organization databases provided 779 publications, of which 12 case-control studies presented the odds of having typhoid fever for those exposed to unimproved or unsafe versus improved drinking water sources. The odds of typhoid fever among those exposed to unimproved or unsafe water ranged from 1.06 to 9.26 with case weighted mean of 2.44 (95% CI: 1.65–3.59). Besides water-related risk, the studies also identified other risk factors related to socioeconomic aspects, type of food consumption, knowledge and awareness about typhoid fever, and hygiene practices.

**Conclusions:**

In this meta-analysis, we have quantified the pooled risk of typhoid fever among people exposed to unimproved or unsafe water which is almost two and a half times more than people who were not exposed to unimproved or unsafe water. However, caution should be exercised in applying the findings from this study in modeling typhoid fever disease burden at country, regional, and global levels as improved water does not always equate to safe water.

## 1. Introduction

Typhoid fever is a systemic bacterial illness of public health importance. The disease is transmitted person to person due to fecal contamination of food and water [1]. The causative agent,* Salmonella enterica *serovar Typhi (*S.* Typhi), is exclusive to humans who are the natural host and reservoirs [2]. Humans can become chronic carriers and food handling practices among carriers can result in food contamination and* S*. Typhi transmission [2]. However, use of sewage contaminated water for irrigation and domestic use is considered critical in maintaining typhoid endemicity in developing countries as demonstrated in Santiago, Chile [2]. Since the major routes of transmission of typhoid fever are through drinking water or eating food contaminated with* Salmonella typhi*, the World Health Organization (WHO) recommends provision of safe water as one of the preventive measures for typhoid fever [2].

Defining and monitoring quality and ensuring water safety in low- and middle-income countries (LMICs) are challenging. The WHO defines microbiologically safe water based on the amount of* Escherichia coli *which should be 0 CFU/100 ml [3] suggesting there should not be any fecal contamination. Continuous monitoring of the microbiologically safe water requires periodic laboratory testing of water sources which is difficult in resource poor settings of LMICs. To simplify the process WHO-UNICEF Joint Monitoring Programme (JMP) has defined alternative indicators, “improved water” and “unimproved water” sources [4], which deemed to represent safe water and unsafe water, respectively (Table 1).

People who drink safe water are likely to have lower risk of typhoid fever compared to people drink unsafe water which is one of the several risk factors for typhoid fever. However, typhoid fever global disease burden estimates often extrapolate the incidence rates obtained from high-risk populations to rest of the populations [5, 6] which is likely to be an overestimation. Hence, it is necessary to correct the incidence rates while extrapolating the data collected from populations drinking unsafe water to population drinking safe water. But, there is no database that provides information on drinking safe water or unsafe water that can be used in global disease burden estimation. Alternately, there is global database available on access to improved water to populations [7] which can be used as a proxy for safe water consumption. Therefore, it is necessary to link the risk of unsafe water to unimproved water. Although a systematic review presented earlier has showed that the microbiological safety of improved water is inconsistent [8] but provides a measure of sanitary protection and it is the only dataset that can be applied at the global level for water-related risk correction.

While many studies have explored the risk of typhoid fever from unsafe water, there has not been a systematic review that presented pooled estimate of the quantitative risk. We conducted a systematic literature review to quantify the probability of symptomatic* S*. typhi infection among residents who consumed unimproved or unsafe water compared to residents who did not consume unimproved or unsafe water. The primary purpose of this review was to derive a quantitative value on excess risk of typhoid fever due to the consummation of unimproved or unsafe water which can be used as a correction factor in global disease burden estimates [6].

## 2. Materials and Methods

A systematic literature review was conducted independently by each of two researchers. Each researcher first identified studies on risk factors for typhoid fever and then selected from those publications, papers presenting water-related risks. The search results from two researchers were compared and any differences between them were resolved based on discussion and agreement. If unresolved, a third independent researcher made the final decision. All selected papers were reviewed by a third researcher before data extraction to confirm its adherence to inclusion and exclusion criteria.

To identify studies, in addition to searching primary databases, PubMed and Embase, searches were also made in WHO and Pan American Health Organization (PAHO) databases. The search was limited to studies published in English language, from January 1, 1990, to December 31, 2013. The detailed inclusion and exclusion criteria are provided in Table 2. The search terms used were (“typhoid” OR “typhoid fever” OR “*Salmonella* Typhi” OR “*S.* Typhi” OR “*Salmonella* infection” OR “enteric fever”) AND (“risk factors” OR “predictive factors” OR “associated factors” OR “attributed factors” OR “exposure factors” OR “related factors” OR “predisposing factors”). Search results are documented in a PRISMA diagram [31].

To quantify the risk, we selected one risk factor from each case-control study that best represented either improved or unimproved water based on the definition provided by WHO/UNICEF-JMP (Table 1). If a water source in a study was identified as “improved,” but was reported to be “microbiologically contaminated,” we considered the water source as “unsafe.” We extracted the odds ratio of typhoid fever among those who got exposed to unimproved water or unsafe water compared to those who did not get exposed. A meta-analysis was conducted to pool the odds ratio of methodologically similar studies using a Metafor Statistical Packages for R, version 1.9-8 [32, 33]. According to the heterogeneity test such as Q statistics and I^2^ [33], the mean value for pooled odds ratio from case-control studies were calculated from a meta-analysis using random effects model with restricted maximum-likelihood estimator [33]. Cohort study findings were descriptively presented as they could not be combined with case-control study meta-analysis. We also descriptively summarized other risk factors that showed a statistically significant probability of symptomatic* S*. typhi infection from the selected studies for the better understanding of overall risk factors.

## 3. Results and Discussion

**Figure 1:**
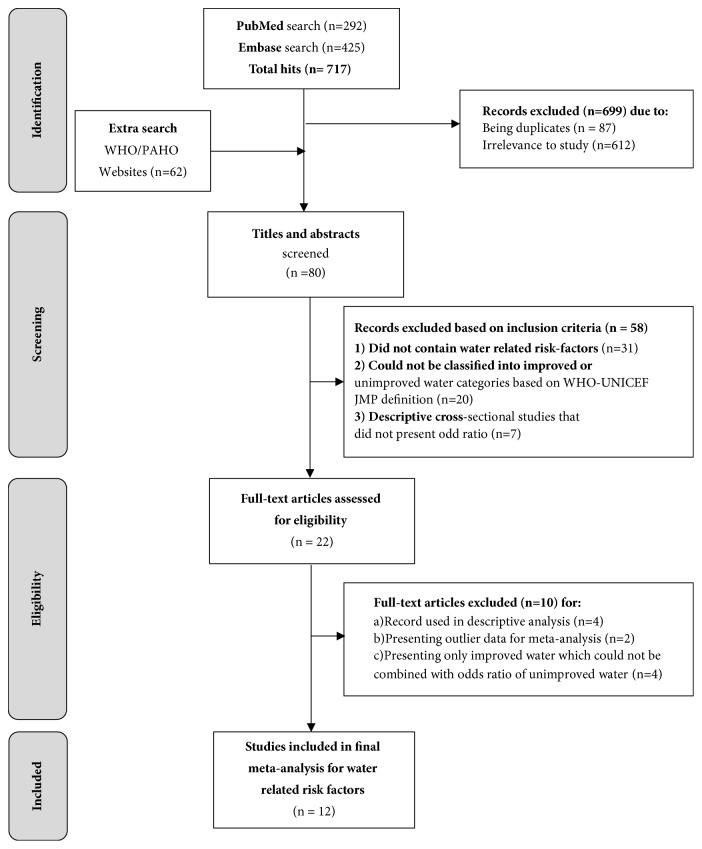
PRISMA diagram representing search results of typhoid fever risk factors.

Our review yielded a total of 779 publications from the search databases (Figure 1). A total of 87 duplicates were removed, and 612 were excluded on title and abstract search because they lacked data on typhoid fever related risk factors. Full texts were accessed for remaining 80 papers. Of them, 58 were excluded as they either (a) did not contain water-related risk factors or (b) could not be classified into improved or unimproved water categories based on WHO-UNICEF-JMP definition or (c) descriptive cross- sectional studies that did not present odd ratio. There were no randomized control trials. Four cohort studies were presented descriptively [27–30] as the risk could not be merged and summarized with majority case-control studies. Finally, we could include only case-control studies in the estimation of pooled odds ratio. Sensitivity analysis was conducted for the decision of study exclusion. At the beginning of the analysis, one study was automatically excluded due to zero-count cell [34] and another one was omitted by sensitivity analysis as an outlier [9]. Four studies had presented odds ratio for improved water which could not be combined with odds ratio for unimproved water because inverse odds of improved water are technically not the same as unimproved water [23–26]. We cannot assume that people unexposed to improved water are exposed to unimproved water. The pooled odds ratio presented below include 12 case-control studies from reported typhoid endemic regions and presented water-related risk factors.

### 3.1. Case-Control Studies

**Figure 2:**
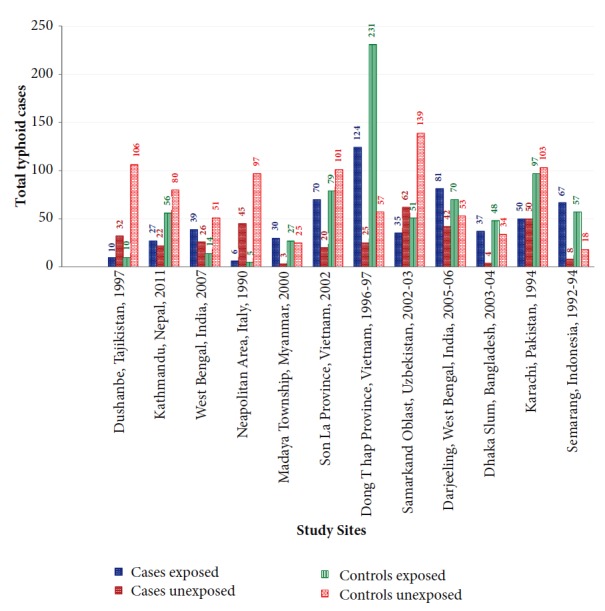
Exposure to unimproved water among typhoid fever cases and controls in selected studies.

**Figure 3:**
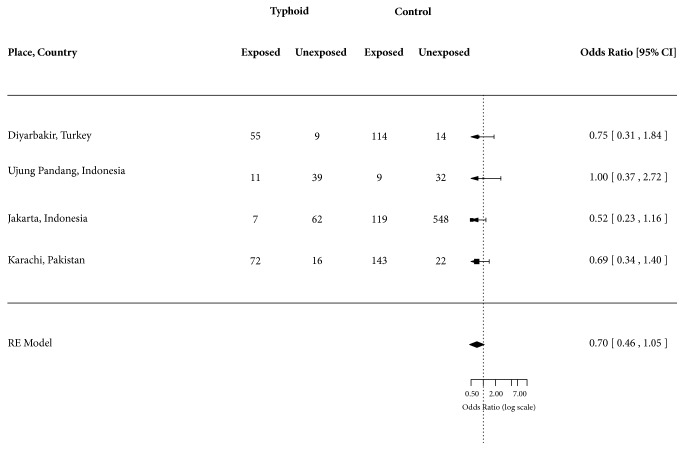
Forest plot showing odds ratio for typhoid fever for exposure and nonexposure to unimproved water.

Of the 12 selected studies, five were from South Asia [12, 13, 19–21], four were from Southeast Asia [15–17, 22], two were from Central Asia [11, 18], and one was from South-Central Europe [14]. The studies included were mostly conducted in urban settings (75.00%) and only three were outbreak investigations (25.00%, Table 3). There were 915 typhoid fever cases and 1,609 nontyphoid fever controls. The exposure to unimproved water was higher among cases (62.95%; n = 576/915) compared to controls (46.30%; n = 745/1609) (Figure 2). Half of the cases-controls studies having improved water source were microbiologically contaminated and were considered unsafe water (Table 3). The odds of typhoid fever among those who were exposed to unimproved water or unsafe water were ranged from 1.06 to 9.26 with case weighted mean of 2.44 (95% CI: 1.65 – 3.59) (Figure 3).

Besides water-related risk, the studies also listed other risk factors related to socioeconomic aspects, living condition, food consumption, knowledge and awareness about typhoid fever, and hygiene practices (Table 4).

### 3.2. Cohort Studies

Four cohort studies presented relative risk of typhoid fever attributable to exposure to unimproved water sources compared to improved water sources [27–30]. The risk of contracting typhoid fever in groups exposed to drinking from a government water supply tank in Rajasthan was 11.10 (95% CI: 3.70 – 33.00) times greater than those in the nonexposed group [27]. Those who drank from combined sources of government tank, hand pump, and personal tube well were 3.75 (95% CI: 1.02 – 13.80) times more likely at risk of typhoid than those not exposed to the three combined sources indicating contamination of these sources. On a floating island restaurant in France, those who drank piped water onboard from untreated River Seine source had no excess risk of typhoid fever compared to those unexposed to those sources (RR = 1.40 95% CI: 0.60 – 3.00) [28]. This study concluded that consumption of rice and chicken washed in tap water resulted in outbreak and found fecal contamination in the tap water which was untreated. In urban Karachi, univariate analysis showed that individuals who consumed tap or bottled water had same risk (RR = 0.70; 95% CI: 0.44 – 1.11) of getting typhoid fever compared to those who did not use tap or bottled water [30]. However, using regression model, after adjusting for all covariates, the study found that overall risk of typhoid fever is lower among households using a safe drinking water source (RR = 0.63; 95% CI: 0.41–0.99). In Eastern Kolkata, the study found that a significantly lower proportion of households use tap water (RR = 0.07; p value = <0.001) in typhoid fever high-risk areas compared to typhoid fever low-risk areas [29].

### 3.3. Case-Control Studies Excluded from Meta-Analysis

**Figure 4:**
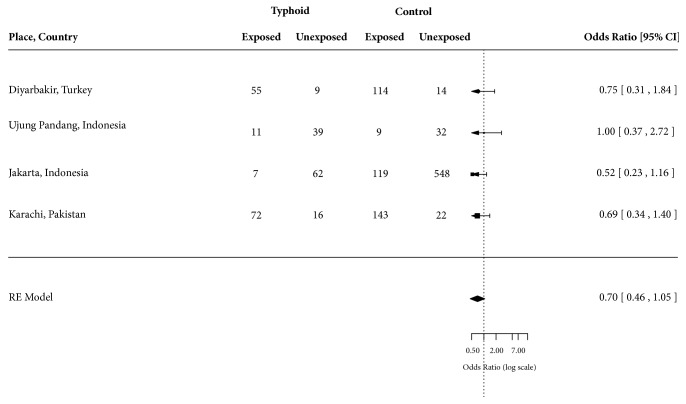
Forest plot showing odds ratio for typhoid fever for exposure and nonexposure to improved water.

The four excluded case-control studies that presented odds of exposure to improved water among typhoid fever cases compared to controls [23–26] when combined together did not show any significant association with water source (OR = 0.70; 95% CI: 0.46 – 1.05) (Figure 4). The risk factors selected from these four studies included utilization of municipal drinking water (OR = 0.75; 95% CI: 0.31 – 1.84) in Diyarbakir, Turkey [23], utilization of piped water (OR = 1.00; 95% CI: 0.37 – 2.72) in Ujung Pandang, Indonesia [24], drinking piped water (OR = 0.52; 95% CI: 0.23 – 1.16) in Jakarta, Indonesia [25], and utilization of tap water (OR = 0.69; 95% CI: 0.34 – 1.40) in Karachi, Pakistan [26].

**Figure 5:**
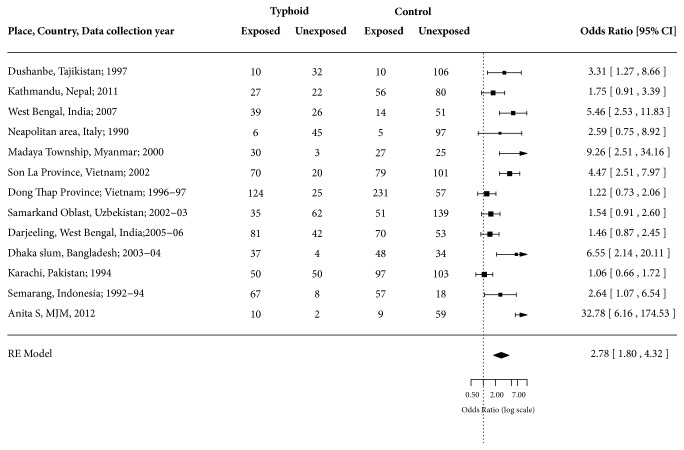
Forest plot showing odds ratio for typhoid fever for exposure and nonexposure to unimproved water after including one outlier study [9].

Two case-control studies excluded from our investigations at the time of sensitivity analysis were from Thailand [34] and Malaysia [9]. In Thailand study, drinking unboiled spring water had 37.80 (95% CI: 1.93 – 739.89) odds of typhoid fever compared to those who drank from either piped water, rain water, commercially bottled water, or water from wells. However, all typhoid fever cases were exposed to unboiled spring water. In Malaysian study, the accidental ingestion of water during swimming or bathing in a river had 32.78 (6.16 – 174.54) odds of getting typhoid fever compared to exposure from food items. In Figure 5 we have presented forest plot for odds ratio without excluding this study to show how its inclusion would have changed the results.  Table 5 presents PRISMA checklist.

### 3.4. Discussion

The systematic review of literature yielded 12 case-control studies from 12 sites conducted in 10 different countries and presenting variables for water-related risk that could be categorized as unimproved water or unsafe water and associated with typhoid fever. This review demonstrates that unimproved water and unsafe water are associated with quantifiable odds of having typhoid fever. The result summary has been used in estimation of typhoid fever disease burden in LMICs [6] which demarcates high-risk population who would benefit maximum from typhoid interventions such as improving water and sanitation or vaccination. Other significant risk factors associated with the occurrence of typhoid fever were related to food consumption, socioeconomic status, hygiene and sanitary practices, living condition, and water storage and handling. These factors should be quantified in future analyses and should be included in future typhoid disease burden estimates. We have not accounted for environmental factors such as rain fall and temperatures, and anthropological measures such as age in this review which should be the other considerations in future disease burden studies.

The importance contaminated water as a major risk factor for typhoid fever is undisputable. During high-endemic period of typhoid fever in Santiago, Chile, the sewage contamination of food chain was demonstrated as the most important factor contributing to typhoid fever transmission, more than the typhoid carrier state in the family members [2]. The past epidemiological studies have demonstrated the importance of waterborne transmission, showing that only small inocula is sufficient for waterborne typhoid transmission, while foodborne transmission requires large inocula [2]. The key role of water and sanitation in typhoid fever transmission is further validated by a correlation between installment of water and sanitation system and decline in typhoid fever cases in industrialized countries. The progressive introduction of water filtration system in later 19th century was correlated with decline in typhoid fever mortality in United States of America (USA) [35]. A more decisive correlation was demonstrated in Philadelphia, USA, where water filtration system was serially introduced in six different districts between 1902 and 1909 [36]. When the condition of water supply and cause specific death rates for various diseases were examined, only typhoid fever deaths were found declining significantly following the introduction of water filtration system.

Although poor water and sanitation system is not the only risk for typhoid transmission, its undisputable importance makes it a key risk factor in defining high-risk groups. Demarcating the typhoid fever risk groups is especially important in effectively targeting control measures such as vaccination programs. The WHO has recommended targeted vaccination of high-risk population with existing typhoid polysaccharide vaccine [1]. The significance of defining high-risk groups has increased with impending availability of typhoid conjugate vaccine [37], which may necessitate revisiting of WHO policies on vaccination strategies based on well-delineated target population. Most surveillance studies were conducted in known typhoid high-risk populations, which cannot be simply extrapolated to general population because their risk of typhoid fever is lower [6, 10]. One of the several risk corrections that can be made in applying the typhoid fever incidence from high-risk population to general population is correct for water-related risk. However, there is no data available at global level of safe water drinking, but there is a database available on improved water and unimproved water [7]. Whereas improved water is representative of safe water and unimproved water is representative of unsafe water, the only available database can be applied at the global level for water-related correction in disease burden estimate. Computing the excess risk associated with the consumption of unsafe water or unimproved water will help in understanding the additional typhoid risk in certain populations and helps in measuring risk-differential typhoid fever incidence in different communities [6]. Such characterization of disease burden that can be linked to access to improved water can help in developing risk-based vaccination strategies and forecasting vaccine demand [38], identifying high-risk populations within countries and targeting vaccination to specific population, estimate its impact, calculate cost-effectiveness, and compare the efficiency of targeted vaccination versus vaccination of whole population.

### 3.5. Limitations

Our study has many limitations. First, we used a basic definition of improved water to represent safe water because this variable is officially reported by WHO-UNICEF-JMP and a global data base is available that can be applied to LMICs in computing risk-differential typhoid fever disease burden. However, improved water does not always equate to safe water in many LMICs [8, 39] and in this paper half of the case-control studies reported microbiological contamination of improved water sources. Although microbiologically unsafe water sources were combined with unimproved water sources to estimate the excess risk of typhoid fever associated with unsafe water, the results may not be generalizable to country levels as this study represented only small number of countries. Similarly, caution should be applied in generalizing the finding to unimproved water as we included both unimproved and unsafe water in one category. Second, evidence from randomized control trials is valued the highest followed by longitudinal prospective cohort studies and case-control studies based on hierarchy of strength of evidence. We had to exclude four prospective cohort studies from meta-analysis approach because it was not possible to integrate the findings from those studies into the analysis. However, these cohort studies have suggested unimproved water as an important risk factor for typhoid fever. Third, it is worth noting that we used only one variable from each case-control study that best matched “unimproved water” to keep the analysis simple. It was challenging to categorize some water sources as improved or unimproved as they did not fit into any category and we had to choose one from the remaining variables. Selection of any other variables may have presented different values or may have resulted in ambiguous findings. Fourth, some of the water sources matched the definition of improved water but a statement from investigators revealed a case of clear contamination of improved water due to reasons such as proximity to sewage pipes and breakage in water supply systems made it necessary to reconsider the improved water as unsafe. This actually deviated from the definition of unimproved water but represented unsafe water which was critical measure for risk differentials. We had club these two categories in our analysis. Fifth, the typhoid fever risk from unsafe water is represented only by 12 studies in our systematic review. Number of studies is too small to generalize and mostly represent Asia. Caution is necessary in the application of results to global disease burden estimation. Sixth, we could have missed some vital papers on water-related risk factors for typhoid fever published in other languages besides English because of search criteria. Also, our search did not include unpublished literature such as conference abstracts, doctoral thesis, or meeting presentations. This may have resulted in publication bias. Lastly, we have used only those papers containing water-related risk factors in our review and, hence, many other significant typhoid fever risk factors outside selected papers may not have been captured in this review. We could have missed some important other risk factors not presented in these studies.

### 3.6. Conclusions

In conclusion, based on literature review we demonstrated that the exposure to unimproved water or unsafe water is significantly associated with typhoid fever. Our findings suggest that the population without access to safe water may be considered as one indicator to delineate high-risk population for typhoid related interventions. The high-risk population decided based on lack of access to safe water can be targeted for typhoid vaccination in addition to ongoing effort to improve water and sanitation infrastructure. Future research should focus on demarcating and quantifying other factors associated with typhoid fever in addition to water-related one, so that more comprehensive risk-association mapping based on geographical information system could be developed and used for targeting typhoid interventions.

## Figures and Tables

**Table 1:** Improved and unimproved drinking water sources based on WHO/UNICEF Joint Monitoring Programme for water supply and sanitation [4].

Improved drinking water source	Unimproved drinking water source
Piped water into dwelling, yard or plot	Unprotected spring
Public tap or standpipe	Unprotected dug well
Tubewell or borehole	Cart with small tank/drum
Protected dug well	Tanker-truck
Protected spring	Surface water
Rainwater collection	Bottled water from unimproved water source^*∗*^

^*∗*^Please refer to WHO/UNICEF Joint Monitoring Programme for water supply and sanitation [4] for details. Note that any microbiologically contaminated water source was considered unsafe water in the analysis.

**Table 2:** Selection criteria for systematic literature review.

**Inclusion criteria**
(i) Publications listed from January 1, 1990 to December 31, 2013
(ii) Studies in English language
(iii) Research conducted in human subjects
(iv) Studies listed in PubMed database or Embase database or WHO website or PAHO website
(v) Study designs: case-control, cohort, randomized control trials
(vi) At least one water related exposure variable that could be categorized either as improved or unimproved drinking water source [4]
(vii) Water is consumed by drinking

**Exclusion criteria**
(i) Descriptive cross sectional studies that did not present odds ratio, case reports and case series
(ii) Studies that did not present water related risk-factors
(iii) Studies conducted in typhoid non-endemic area are excluded in the estimation of pooled odds ratio

**Table 3:** Characteristics of case-control and cohort studies included in the systematic literature review.

Publication year	Study year	Study site	Study setting	Site population	Selected unimproved/unsafe water source	Reasons for typhoid fever	Source
Case control studies							
1999	1997	Dushanbe, Tajikistan	Outbreak in endemic area	Urban	Water for home obtained from outside tap [10] (contaminated improved source = unsafe)	Water source contamination Cessation of chlorination, intermittent water supply creating negative pressure and contaminating water supply with surrounding contaminants.	[11]
2013	2011	Kathmandu, Nepal	Endemic	Urban	Use of stone spout water	Multiple; drinking water spout contamination with sewage, contamination of stored water, general sanitation issues such as lack of toilets or lack of water to flush toilets	[12]
2009	2007	West Bengal, India	Outbreak in endemic area	Urban slum	Drinking piped water [10] (contaminated improved source = unsafe)	Unchlorinated water supply through pipes, drinking water pipes close to open drainage and intermittent water supply	[13]
1992	1990	Neapolitan Area, Italy	Outbreak in endemic area	Urban	Drinking non-potable water	Multiple; foodborne, sanitation, and drinking water source contamination due to sewage exposure to municipal water supply	[14]
2004	2000	Madaya Township, Myanmar	Outbreak in endemic area	Rural	Drinking untreated river water	Drinking water contamination with unchlorinated river water which had direct sewage drainage.	[15]
2005	2002	Son La Province, N. Vietnam	Endemic	Urban	Drinking untreated water	Drinking water contamination and consumption of unchlorinated water (dislike for chlorine smell)	[16]
2005	1996-1997	Dong Thap Province, Mekong Delta, S. Vietnam	Endemic	Urban	Drinking unboiled water	Multiple; drinking river water which had sewage (latrine) drainage, drinking water sources from deep wells and ponds contaminated with drainage from latrines situated in the proximity	[17]
2007	2002-2003	Samarkand Oblast, Uzbekistan	Endemic	Urban and Rural	Consumption of unboiled surface water outside the home	Water source contamination. The drinking of un-boiled surface water outside home during the hot and dry summer months.	[18]
2009	2005-2006	Darjeeling, West Bengal, India	Endemic	Rural	Stream water	Multiple; foodborne, sanitation issues, and drinking water source contamination. Untreated water supply from unprotected springs and natural streams, untreated water supply from venders.	[19]
2007	2003-2004	Dhaka slum, Kamalapur, Bangladesh	Endemic	Urban slum	Drinking unboiled water at home	Multiple; sanitation issues, and drinking water source contamination. Partial chlorination of municipal water supply exposed to contamination, drinking of untreated water	[20]
1998	1994	Karachi, Pakistan	Endemic	Urban	Drinking water at work (improved or unimproved unknown)	Multiple; foodborne, sanitation issues, and drinking water source contamination at workplace	[21]
2001	1992-1994	Samarang, Indonesia	Endemic	Urban	Drinking non-municipal water source	Multiple; foodborne, sanitation issues, and drinking of unchlorinated water from venders	[22]
2005	2001-2003	Diyarbakir, Turkey	Endemic	Urban & rural	Municipality drinking water (contaminated improved water source = unsafe)	Consumption of raw vegetables irrigated with sewage water from the city	[23]
1997	1990-1991	Ujung Pandang, Indonesia	Endemic	Urban	Piped water (contaminated improved water source = unsafe)	Street food consumption	[24]
2004	2001-2003	Jakarta, Indonesia	Endemic	Urban	Piped water (contaminated improved water source == unsafe)	Hygienic practices such as no use of soap for handwashing, sharing of food, and no toilet in the household and household crowding at home	[25]
2008	1999-2001	Karachi, Pakistan	Endemic	Urban	Piped water (contaminated improved water source = unsafe)	Hygienic practices such as lack of soap availability at handwashing place, frequently eating outside home and crowing at home	[26]

Cohort studies							
2010	2007	Rajasthan, India	Outbreak in endemic area	Rural	Drinking water from government tank, hand pump and personal tube well	Contaminated sources due to an open well supplying water to all the three water supply facilities	[27]
2000	1998	River Seine, Paris, France	Outbreak	Urban	Drinking untreated river water	Fecal contamination of tap water	[28]
2007	2003 – 2004	Eastern Kolkata, India	Endemic	Urban	Drinking unsafe drinking water	NA	[29]
2012	2003 – 2006	Karachi, Pakistan	Endemic	Urban	Drinking tap water	NA	[30]

NA – Not provided.

**Table 4:** Other significant risk factors for typhoid fever identified in reviewed papers.

Study Site	Risk Category	Exposure factor	AOR (95% CI; p-value if any)	Source
Dushanbe, Tajikistan	Water storage and handling	Unboiled water	6.5 (3.0 – 24.0; p < 0.05)^*∗*^	[11]
	Food handling and consumption	Eating food obtained from street vendor	2.9 (1.4 – 7.2; p < 0.05)^*∗*^	

Kathmandu, Nepal	Socio-economic	Household monthly income < US$125	2.55 (1.1 – 6.0; p < 0.05)	[12]
	Hygiene & sanitary	Household latrine	8.52 (1.8 – 40.1; p < 0.05)	
	Water storage & handling	Water stored after collection	3.09 (1.2 – 8.1; p < 0.05)	
		Eaten street food < 2 weeks	2.34 (1.4 – 4.0; p < 0.05)	

West Bengal, India	Food handling and consumption	Food from sweet shop	6.2 (2.4 – 2.2)^*∗∗*^	[19]
	Socio-economic	Monthly family income < 1500	6 (1.3 – 26.8)	
		INR (eqvt. US$ 34)		
	Living condition	Household > 4 members	4.2 (1.7 – 11.1)	
	Living condition	Anyone ill in neighborhood	2.5 (1.2–5.2)	
	Food handling and consumption	Eating of Paratha (flatbread in layers)	2.1 (0.87–5.3)	

Neapolitan Area, Italy	Food handling and consumption	Any raw Shellfish	13.3 (5.5 – 32.8; p < 0.05)^*∗∗*^	[14]
	Food handling and consumption	Raw Oysters	9.3 (1.7 – 67.3; p < 0.05)^*∗∗*^	
	Food handling and consumption	Raw Mussels	8.9 (3.9 – 21.1; p < 0.05)^*∗∗*^	
	Food handling and consumption	Raw Hen Clams	8.1 (2.8 – 23.7; p < 0.05)^*∗∗*^	
	Food handling and consumption	Raw Sea Truffles	6.4 (1.4 – 32.9; p < 0.05)^*∗∗*^	

Madaya Township, Myanmar	Living condition	Contact with typhoid patient	10.9 (2.0-79.7)	[15]

Son La Province, Northern Vietnam	Socio-economic	Being uneducated	2.0 (1.0 – 3.7; p = 0.03)	[16]
	Living condition	Contact with typhoid patient	3.3 (1.7 – 6.2; p < 0.05)	

Samarkand Oblast, Uzbekistan	Socio-economic	Student as primary occupation	4.0 (1.4 – 11.3; p < 0.05)	[18]
		Taken antimicrobials in 2 weeks before illness onset	12.2 (4.0 – 37.0; p < 0.05)	

Darjeeling, West Bengal, India	Food handling and consumption	Eating raw cabbage	2.8 (1.7-4.8)^*∗*^	[19]
	Water storage & handling	Scooping water from a container with a cup	2.5 (1.3-4.7)^*∗*^	
	Food handling and consumption	Consumption of butter	2.3 (1.3-4.1)^*∗*^	
	Food handling and consumption	Consumption of Yoghurt	2.3 (1.4-3.7)^*∗*^	
	Food handling and consumption	Eating unwashed grapes	2.2 (1.3-4.0)^*∗*^	
	Food handling and consumption	Eating raw onion	2.1 (1.2-3.9)^*∗*^	
	Food handling and consumption	Eating raw carrot	2.1 (1.2-3.9)^*∗*^	

Dhaka slum, Kamalapur, Bangladesh	Water storage & handling	Consumption of foul-smelling water	7.4 (2.1 – 25.4; p = 0.002)	[20]
	Food handling and consumption	Consumption of unwashed Papaya	5.2 (1.2 – 22.2; p = 0.03)	

Karachi, Pakistan		Taken antimicrobials in 2 weeks prior to illness	3.0 (1.4 – 6.5)	[21]
	Food handling and consumption	Eating at restaurant between July and August	2.7 (1.1-6.6; p = 0.01)^*∗*^	
	Food handling and consumption	Eating at road side cabin between July and August	2.4 (1.0-5.6; p = 0.03)^*∗*^	
	Food handling and consumption	Eating out >1 per week between July and August	2.3 (1.0-5.2; p = 0.02)^*∗*^	
	Food handling and consumption	Eating ice cream	1.7 (1.0-3.1; p = 0.03)^*∗*^	
	Food handling and consumption	Eating a commercial brand of ice cream	1.6 (1.0-2.9; p = 0.04)^*∗*^	

Semarang, Indonesia	Hygiene & sanitation	Never or sometimes washing hands before eating	3.97 1.22-12.93; p = 0.022)	[22]
	Hygiene & sanitation	Open sewage system or no drainage system in house	7.19 (1.33-38.82; p = 0.022)	
	Socio-economic	Being unemployed or part-time jobber	31.3 (3.08-317.4; p = 0.036)	

Mekong Delta, Vietnam	Living condition	Recent contact with typhoid fever case	4.3 (1.4-13.4; p = 0.04)	[17]
	Socio-economic	Low economic level	2.5 (1.3-5.1; p = 0.01)	

River Seine, Paris, France		Eating chicken on boat	2.0 (0.3 – 13.7)^#^	[28]
		Eating rice on boat	2.9 (0.4 – 19.8)^#^	

Eastern Kolkata, India		Using latrine in-house latrine for defecation	5.32 (p = 0.9)	[29]

Karachi, Pakistan		Living in densely populated area	2.43 (1.27 – 4.64; p = 0.01)	[30]

^∧^Frozen mamey pulp imported from Guatemala used in fruit shake

^∧∧^Cig Kofte is a traditional raw food made from raw meat rolled in a ball form

^*∗*^MOR – Matched odds ratio

^*∗∗*^OR – Odds ratio

^#^RR – Relative risk ratio.

**Table 5:** PRISMA 2009 checklist.

Section/topic	#	Checklist item	Reported on page #
**TITLE **			

Title	1	Identify the report as a systematic review, meta-analysis, or both.	Cover page Abstract 2, 3

**ABSTRACT **			

Structured summary	2	Provide a structured summary including, as applicable: background; objectives; data sources; study eligibility criteria, participants, and interventions; study appraisal and synthesis methods; results; limitations; conclusions and implications of key findings; systematic review registration number.	Abstract

**INTRODUCTION **			

Rationale	3	Describe the rationale for the review in the context of what is already known.	1

Objectives	4	Provide an explicit statement of questions being addressed with reference to participants, interventions, comparisons, outcomes, and study design (PICOS).	1

**METHODS **			

Protocol and registration	5	Indicate if a review protocol exists, if and where it can be accessed (e.g., Web address), and, if available, provide registration information including registration number.	NA

Eligibility criteria	6	Specify study characteristics (e.g., PICOS, length of follow-up) and report characteristics (e.g., years considered, language, publication status) used as criteria for eligibility, giving rationale.	2, 16

Information sources	7	Describe all information sources (e.g., databases with dates of coverage, contact with study authors to identify additional studies) in the search and date last searched.	2

Search	8	Present full electronic search strategy for at least one database, including any limits used, such that it could be repeated.	2,Table 2

Study selection	9	State the process for selecting studies (i.e., screening, eligibility, included in systematic review, and, if applicable, included in the meta-analysis).	2, Table 2

Data collection process	10	Describe method of data extraction from reports (e.g., piloted forms, independently, in duplicate) and any processes for obtaining and confirming data from investigators.	2, Figure 1

Data items	11	List and define all variables for which data were sought (e.g., PICOS, funding sources) and any assumptions and simplifications made.	9, Table 3

Risk of bias in individual studies	12	Describe methods used for assessing risk of bias of individual studies (including specification of whether this was done at the study or outcome level), and how this information is to be used in any data synthesis.	NA

Summary measures	13	State the principal summary measures (e.g., risk ratio, difference in means).	3, 16

Synthesis of results	14	Describe the methods of handling data and combining results of studies, if done, including measures of consistency (e.g., I^2^) for each meta-analysis.	2, 3 Figure 3

Risk of bias across studies	15	Specify any assessment of risk of bias that may affect the cumulative evidence (e.g., publication bias, selective reporting within studies).	NA

Additional analyses	16	Describe methods of additional analyses (e.g., sensitivity or subgroup analyses, meta-regression), if done, indicating which were pre-specified.	NA

**RESULTS **			

Study selection	17	Give numbers of studies screened, assessed for eligibility, and included in the review, with reasons for exclusions at each stage, ideally with a flow diagram.	Figure 1

Study characteristics	18	For each study, present characteristics for which data were extracted (e.g., study size, PICOS, follow-up period) and provide the citations.	Table 3

Risk of bias within studies	19	Present data on risk of bias of each study and, if available, any outcome level assessment (see item 12).	NA

Results of individual studies	20	For all outcomes considered (benefits or harms), present, for each study: (a) simple summary data for each intervention group (b) effect estimates and confidence intervals, ideally with a forest plot.	Figures 3 and 4

Synthesis of results	21	Present results of each meta-analysis done, including confidence intervals and measures of consistency.	Figures 3 and 4

Risk of bias across studies	22	Present results of any assessment of risk of bias across studies (see Item 15).	NA

Additional analysis	23	Give results of additional analyses, if done (e.g., sensitivity or subgroup analyses, meta-regression [see Item 16]).	Figure 4

**DISCUSSION **			

Summary of evidence	24	Summarize the main findings including the strength of evidence for each main outcome; consider their relevance to key groups (e.g., healthcare providers, users, and policy makers).	8

Limitations	25	Discuss limitations at study and outcome level (e.g., risk of bias), and at review-level (e.g., incomplete retrieval of identified research, reporting bias).	7, 8

Conclusions	26	Provide a general interpretation of the results in the context of other evidence, and implications for future research.	8, Figure 2

**FUNDING **			

Funding	27	Describe sources of funding for the systematic review and other support (e.g., supply of data); role of funders for the systematic review.	9

From [31].

## Data Availability

All data related to the research is available in the manuscript.

## Abbreviations

*S.* Typhi: * Salmonella enterica* serovar Typhi
WHO: World Health Organization
LMICs: Low- and middle-income countries
CFU: Colony forming units
UNICEF: United Nations International Children Emergency Relief Fund
JMP: Joint Monitoring Programme
PAHO: Pan American Health Organization
RR: Relative risk
OR: Odds ratio
USA: United States of America.
